# Effect of night-shift work on cortisol circadian rhythm and melatonin levels

**DOI:** 10.5935/1984-0063.20220034

**Published:** 2022

**Authors:** Maria Carlota Borba Brum, Martha Bergan Senger, Claudia Carolina Schnorr, Lethicia Rozales Ehlert, Ticiana da Costa Rodrigues

**Affiliations:** 1 Clinical Hospital of Porto Alegre, Division of Occupational Medicine - Porto Alegre - Rio Grande do Sul - Brazil.; 2 Clinical Hospital of Porto Alegre, Division of Clinical Pathology - Porto Alegre - Rio Grande do Sul - Brazil.; 3 Clinical Hospital of Porto Alegre, Division of Peripheral Vascular Surgery - Porto Alegre - Rio Grande do Sul - Brazil.; 4 Clinical Hospital of Porto Alegre, Division of Endocrinology - Porto Alegre - Rio Grande do Sul - Brazil.; 5 Federal University of Rio Grande do Sul, School of Medicine, Department of Internal Medicine - Porto Alegre - Rio Grande do Sul - Brazil.

**Keywords:** Cortisol, Melatonin, Night Work, Circadian Rhythm, Desynchronization

## Abstract

**Objectives:**

Night-shift work has been associated with several negative effects on worker’s health, possibly due to circadian desynchronization, sleep deprivation and suppression of nocturnal melatonin secretion including exposure to light during the work shift. The objective of this study was to evaluate the impact of fixed night-shift work versus day-shift work on the sleep-wake cycle and on the night and day levels of cortisol and melatonin.

**Material and Methods:**

Saliva samples were obtained from 36 individuals, 19 day workers (12 women and 7 men) and 17 night workers (12 women and 5 men) from a university hospital in southern Brazil, with no history of chronic diseases. Demographic and personal information were obtained through a self-administered questionnaire and sleep information by the Munich chronotype questionnaire.

**Results:**

Salivary cortisol showed normal circadian rhythm in day- and night-shift workers, but was attenuated in night-shift workers during their working hours and on leave days. Night workers sleep fewer hours at night and have higher negative social jet lag than day workers.

**Conclusion:**

Intervals between night shifts can be beneficial for the recovery of the hypothalamic-pituitary-adrenal axis, minimizing the negative effects on workers’ health, in addition to a preventive approach to aspects related to sleep hygiene and healthy life habits.

## INTRODUCTION

Shift work is associated with several negative effects, including sleep disorders, fatigue, reduced alertness, cognitive deficits, and increased accident rates^1^. In addition, it is implicated in higher rates of metabolic disorders and cardiovascular diseases^2-5^.

The melatonin and cortisol hormones are intrinsically related to the circadian cycle. Melatonin is produced by the pineal gland and released directly into the bloodstream, a process controlled by the hypothalamus’ the suprachiasmatic nucleus (SCN). The concentration peak occurs between 9:00 p.m. and 7:00 a.m., with large individual variation^6^. Its production is controlled by the light-dark cycle; exposure to light blocks its secretion. There is no storage of melatonin in the body and, therefore, its plasma levels reflect pineal activity^7,8^. The main role of melatonin is to act as an endogenous synchronizer of central and peripheral tissues^7^. In addition to its fundamental role in promoting sleep, melatonin has antioxidant, oncostatic, anti-apoptotic and immunomodulatory effects^8^. The pattern of melatonin secretion in plasma, saliva or urine is the best peripheral marker of central oscillator entrainment in humans^6^.

Cortisol is an excellent marker of circadian rhythm^9^. It is controlled by the hypothalamic-pituitary-adrenal (HPA) axis and has anti-inflammatory, metabolic and immunosuppressive effects^1,10^. Cortisol levels usually peak in the morning and decrease throughout the day; concentrations are minimal at night. This cycle has what is known as response to cortisol awakening (CAR), a peak in cortisol production that occurs 20 to 30 minutes after awakening^10^. Factors that influence cortisol secretion include daytime rhythm, alertness and the sleep-wake cycle, along with neural pressure signs^1^. Results showed that the use of salivary cortisol is similar to blood levels, it is easier, painless and can be collected anywhere^11^.

These patterns occur when the circadian system is normally entrained to the 24-h light-dark cycle and are altered or not present in shift workers, those who travel across time zones or in blind individual who have lost intrinsically photosensitive retinal ganglion cells signaling^12^.

Changes in the light-dark synchronization due to continuous exposure to light can contribute to a loss of synchronization of the biological clock. Evidence suggests that circadian desynchronization, sleep deprivation and suppression of nocturnal melatonin release by exposure to light are the main pathological mechanisms through which night-shift work produces harmful effects on worker’s health^13^.

Light exposure at night suppresses melatonin secretion rapidly (5-15 minutes) after the light pulse is initiated, as well as recovery at the end, with short duration (intermittent) stimuli being more effective for recovery than long-term (continuous). The suppressive effects of light exposure on melatonin secretion take place rapidly, whereas recovery is a relatively slower process. Regarding cortisol, this is a more complex mechanism, with an increase in cortisol levels in the first minutes of light exposure, followed by an increase and stabilization at lower levels maintained for a few hours after the exposure ends^14,15^.

The objective of this study was to evaluate the impact of fixed night-shift work versus day-shift work on the sleep-wake cycle and on the night and day levels of cortisol and melatonin.

## MATERIAL AND METHODS

### Study participants and design

Thirty-six healthy workers, of both sexes, from administrative, maintenance and care areas of a university hospital in southern Brazil participated in this study. Nineteen participants were on fixed day shift (8:00 a.m. to 6:00 p.m.), Monday to Sunday with rests on Saturday and Sunday, and 17 participants worked on fixed night shift (7:00 p.m. to 7:00 a.m. with 60 hours rests). Pregnant women; individuals over 60 years old; patients with diabetes, hypertension, dyslipidemia, chronic liver, kidney, or thyroid disease; severe sleep disorders; with a history of drug or alcohol abuse in the last 12 months; on corticosteroid or antiarrhythmic therapy and active periodontal disease that can contaminate saliva collection with blood were excluded from the study.

A self-administered questionnaire was used to collect sociodemographic, occupational and personal information. Health status was assessed by physical examination with weight, height, blood pressure, heart rate (HR) and waist circumference (WC) measurements.

This research was carried out in accordance with the recommendations established by the declaration of Helsinki and approved by the institution’s ethics committee and approved by the ethics committee number 1.317.729.

### Anthropometric indicators and body composition

Weight was measured on a Welmy R/1W-200 digital scale (200kg capacity, 0.100g resolution). Height was measured with a Tonelli stadiometer, at a millimeter scale, with accuracy of 0.1cm. BMI was calculated using the formula: weight (kg)/height^2^ = BMI. Circumferences were taken with a metal measuring tape, accurate to 0.1cm, and maximum length of 2m. Waist circumference (WC) was measured halfway between the lower margin of the last rib and the ilium crest in the horizontal plane. Blood pressure was measured in both arms using the Welch Allyn 300 Series Digital Vital Signs Monitor.

### Chronotype

The chronotype and sleep preferences were assessed by the self-reported Munich chronotype questionnaire (MCTQ), developed and validated in Germany and Switzerland by

Roenneberg et al. (2003)^16^, we used the validated version in Brazil por Alam (2012)^17^ in relation to working days and days off. The data were used to calculate sleep duration and the midpoint of sleep for work and break days, as well as social jet lag, defined as the difference between the respondents’ “biological clock” and their “social clock”, i.e., their social commitments during the day, calculated as the difference between the midpoint of sleep on working days and the midpoint of sleep on break days^18^.

### Cortisol and melatonin sampling

Cortisol and melatonin were measured in saliva, Salicaps^®^ collection tubes (IBL Hamburg, Germany)^19^. On working days, day- and night-shift workers collected saliva samples between 10 p.m. and midnight and between 6 a.m. and 8 a.m. On days off, two saliva samples were collected from night workers between 10 p.m. and midnight and between 6 a.m. and 8 a.m. on the day following the work day. The samples were collected in duplicates, and the levels of cortisol and melatonin were taken as the mean of the two samples collected in each shift. The workers were instructed to collect saliva samples after fasting for 30 minutes or after brushing their teeth; collect in a sitting or reclining position and in low light, away from direct light sources. After collection, the Salicaps^®^ were stored in the participants’ residential freezers and transported to the study facilities in polystyrene foam coolers. Upon receipt by the investigator, the samples were stored at-80 °C. The melatonin and cortisol concentrations in the saliva samples were measured by the Enzyme-Linked Immunosorbent Assay (ELISA) test kit provided by IBL-Hamburg.

For sample size calculation, considering the large interpersonal variability in these biomarkers, the sample size was calculated according to the results of Reinhardt et al. (2013)^20^ in melatonin levels upon waking between day and night workers, with 5% significance and 80% power, resulting in 36 workers.

### Statistical analysis

Quantitative variables were expressed as mean and confidence intervals (CI) and qualitative categorical variables were expressed as absolute and relative frequencies. The chisquare test and Fisher’s exact test were used as appropriate for comparisons between groups of qualitative variables. The Shapiro-Wilk test was used to assess the normality of the distribution. The analyses of quantitative variables were performed using the bootstrapping method and bias-corrected and accelerated bootstrap (BCa) confidence intervals (CI) were obtained with 1,000 bootstrap resamples. The main advantage to the BCa interval is that it corrects for bias and skewness in the distribution of bootstrap estimates^21^. To compare morning and night cortisol and melatonin levels in day-shift, night-shift and night-shift workers on days off, we applied Student’s t-test for paired data with the Bonferroni correction adjustment for multiplicity, setting the significance level for CI estimates for individual level α=0.017 (98.3%CI). Differences between dayshift and night-shift workers in the delta cortisol (daytime sample - nighttime sample), delta melatonin (daytime sample - nighttime sample) variables and sleep-related variables were analyzed with Student’s t-test (raw result) and ANCOVA to adjust for alcohol consumption (adjusted result). The significance level for bootstrapping CI estimates was α=0.05 (95%CI). All statistical analyses were performed in the Statistical Package for the Social Sciences (SPSS) version 23 software environment (IBM Corp., Armonk, NY).

## RESULTS

The study sample consisted of 19 day workers (12 women and 7 men) and 17 night workers (12 women and 5 men), with mean age of 45 years. Day workers had a significantly higher prevalence of alcohol consumption (77.8% vs. 17.6%, *p*=0.001). Health professionals were more prevalent among night-shift workers, while administrative workers were more prevalent in the day-shift group (Table 1). Physical evaluation evaluation showed no differences between shifts.

**Table 1:** Bivariate analysis of demographic, occupational, and lifestyle variables of workers in southern Brazil, (n=36).

Variables	Day-shift	Night-shift	*p*-value
**Age, mean (95%CI)**	44.05 (39.84; 48.27)	46.18 (42.58; 49.68)	0.468*
**Gender, n(%)**			0.906^##^
Female	12 (63.2)	12 (70.6)	
Male	7 (36.8)	5 (29.4)	
**Race, n(%)**			0.092^¥^
Caucasian	13 (68.4)	16 (94.1)	
Others	6 (31.6)	1 (5.9)	
**Working shift (years), mean (95%CI)**	9.94 (4.98; 15.06)	10.73 (7.61; 14.44)	0.805*
**Educational level, n(%)**			0.681^#^
Secondary education	5 (26.3)	6 (35.3)	
Graduate/postgraduate education	9 (47,4)	9 (52,9)	
**Smoking, n(%)**			0.486^¥^
Yes	0 (0.0)	1 (5.9)	
No	18 (100.0)	16 (94.1)	
**Alcohol consumption, n(%)**			**0.001^##^**
Yes	**14 (77.8**)	3 (17.6)	
No	4 (22.2)	14 (82.4)	
**Role, n(%)**			**0.020^#^**
Administrative	**7 (36.8)**	1 (5.9)	
Health care workers	10 (52.6)	**16 (94.1)**	
Maintenance	2 (10.5)	0 (0.0)	

Notes:

* Student t-test 95%CI obtained with 1,000 bootstrap resamples; #Pearson chi-square test; ##Pearson chi-square test with correction for continuity; ¥ Fisher’s exact test.

**Figure 1:**
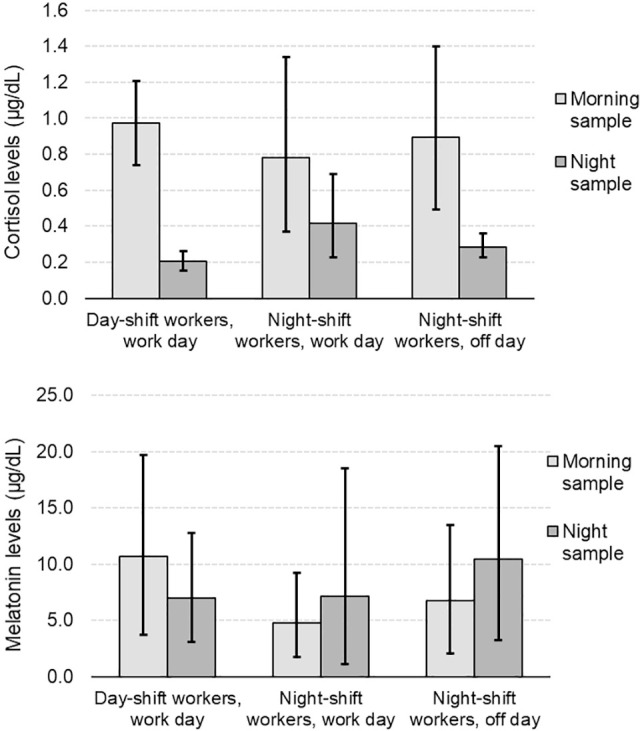
Analysis of cortisol collection times and melatonin levels (µg/dL). Paired Student t-test with Bonferroni correction: 98.3%CI obtained with 1,000 bootstrap resamples.

In daytime workers, we observed a significant decline of cortisol levels in the nighttime samples compared to the morning. Night-shift workers showed a significant difference between cortisol levels in night samples and morning samples at the end of the shift. On days off, cortisol levels were also lower in night samples than in morning samples (Figure 1).

When comparing cortisol levels in the morning vs. at night, a significant difference was seen in day-shift workers (0.97%CI: 0.74; 1.21 vs. 0.21%CI: 0.15; 0.26) and night-shift workers on days off (0.89%CI: 0.49; 1.40 vs. 0.29%CI: 0.23; 0.36), with nighttime levels being lower. In night-shift workers, the observed difference was not significant because the CI limits for morning and night overlap. The same occurred with melatonin and no evidence of significant difference was found between the morning and night measurements in dayshift workers (10.69%CI: 3.69; 19.67 vs. 7.01%CI: 3.05; 12.78), in night-shift workers (4.73%CI: 1.77; 9.19 vs. 7.16%CI: 1.14; 18.55) and night-shift workers on days off (6.71%CI: 2.09; 13.49 vs. 10.47%CI: 3.21; 20.45) (Figure 1).

Sleep duration was significantly shorter in night-shift workers than in day-shift workers (03:68 [3:07-4:41] vs. 06:66 [6:22-7:15], *p*=0.000) on working days, and the sleep midpoint also differed significantly (11:16 [9:84-12:14] vs. 02:98 [2:643:40], *p*=0.000). On days off, no significant difference was seen in sleep duration or sleep midpoint between day- and night-shift workers, however, when adjusted for alcohol consumption, a difference was observed, with night-shift workers sleeping fewer hours in comparison to day-shift workers on days off (9:20 [8:40-10:17] vs. 06:97 [5:68-8:14], *p*=0.003). Regarding jet lag, day- and night-shift workers differed significantly both in the crude and alcohol-consumption adjusted analysis, in which night-shift workers showed negative social jet lag (Table 2). Cortisol and melatonin variations were not different between groups.

**Table 2:** Analysis of delta cortisol, delta melatonin and sleep-related variables between day-shift and night-shift workers in southern Brazil (n=36).

Variables	Day-shift	Night-shift	*p*-value
**Sleep duration, working day** Raw	6.66 (6.22; 7.15)	3.68 (3.07; 4.41)	**0.000**
Adj	6.42 (5.91; 6.87)	3.94 (3.32; 4.65)	**0.000**
**Midpoint of sleep, working day**			
Raw	2.98 (2.64; 3.4)	11.16 (9.84; 12.14)	**0.000**
Adj	3.47 (2.79; 4.8)	10.74 (8.84; 12.18)	**0.000**
**Sleep duration, free day**			
Raw	8.65 (8.03; 9.35)	7.57 (6.61; 8.4)	0.072
Adj	9.2 (8.4; 10.17)	6.97 (5.68; 8.14)	**0.003**
**Midpoint of sleep, free day**			
Raw	4.07 (3.7; 4.44)	4.3 (3.55; 5.00)	0.554
Adj	4.26 (3.73; 4.73)	4.19 (3.39; 4.87)	0.896
**Jet lag** Raw	>1.17 (0.64; 1.59)	-6.21 (-8.08;-3.76)	>**0.000**
Adj	0.97 (-0.21; 1.74)	-6 (-7.83;-3.89)	0.000
**Delta cortisol** Raw	>0.76 (0.55; 1)	0.37 (-0.03; 0.81)	>0.121
Adj	0.56 (0.32; 0.8)	0.56 (0.16; 1.01)	0.986
**Delta melatonina** Raw	>3.68 (-3.33; 11.39)	-2.42 (-10.02; 3.02)	>0.249
Adj	6.91 (-1.15; 17.63)	-5.18 (-14.65; 2.38)	0.075

Notes: Raw: Results by Student t-test; Adj: Results by ANCOVA adjusted by alcohol; 95%CI obtained with 1,000 bootstrap resamples.

## DISCUSSION

Day-shift workers showed salivary cortisol levels as expected, with a higher cortisol level in the morning followed by a decline in the evening. Night-shift workers showed a similar result but at lower thresholds, even on days off, demonstrating that the body tries to maintain physiological cadence.

Niu et al. (2015)^22^ found that the cortisol secretion pattern in night-shift workers returns to the levels of day-shift workers on the second day off after five consecutive night shifts, which means that women who work at night would benefit from being away from work for more than 2 days between shifts to restore the circadian rhythm of daytime cortisol. Similar results were found in our study when comparing working days and days off, and these informations have important impact when think about schedules/shifts for night workers.

In a field study that compared the transition from night shifts to rotating shifts, cortisol levels were found to be low during night shifts, remaining unchanged when switching to rotating shifts. Among day-shift workers who switched to a permanent night-shift, cortisol levels were reduced and tended to normalize after an adaptation period^23^.

Regarding melatonin levels, our results presented not significant difference in this sample. Melatonin levels are not only influenced by the light-dark cycle phases, but also by variations in the intensity and history of light exposure^24^; and even the normal range of melatonin levels can vary substantially between individuals^25^.

A study in mine operators who worked 7-day and 7-night shifts followed by 7 days off, the time of onset of melatonin secretion in saliva changed significantly over the week during the day and night shifts (09:04 p.m. ± 16min vs. 09:30 p.m. ± 16min, respectively), but the small magnitude of the change indicated a lack of adaptation of the true circadian rhythm to this change pattern^26^.

Adaptation of the circadian system to nighttime work depends on a wide range of factors, including light exposure, environmental conditions, wake-up time, work start and end times and individual characteristics^26^.

In our study, we observed that night-shift workers sleep 6 hours less on workdays than day-shift workers, a number of hours of sleep lower than that recommended for adults by the National Sleep Foundation^27^, which characterizes a situation of chronic sleep deprivation as demonstrated by the negative social jet lag in the night-shift group. Cumulative sleep deprivation is often associated with work patterns involving night or morning shifts^26^.

Reinhardt et al. (2018)^28^ compared subgroups of day- and night-shift workers with or without sleep restriction (<6 hours or >6 hours) and demonstrated that night-shift workers without sleep restriction showed slightly lower melatonin levels during the work shift while sleep-restricted night-shift workers showed slightly higher concentrations after awakening, however, such difference was not significant. Similarly, a comparison of nonsleep-restricted and sleep-restricted day-shift workers showed that sleep-restricted day-shift workers secreted significantly less melatonin before bedtime. Light exposure is known to decrease melatonin secretion and delay the melatonin phase in humans^29^.

According to Boivin et al. (2014)^30^, night-shift workers who sleep during the day experience circadian desynchronization similar to that of travelers who quickly cross multiple time zones. However, in night-shift workers, circadian adaptation is more problematic, since individuals remain exposed to exogenous daytime stimuli, including home and family activities while some work double shifts. Cortisol levels during daytime sleep in shift workers are higher than those measured during nighttime sleep in daytime workers^12^.

Chronodisruption is the prolonged impairment of physiological, behavioral and biochemical rhythms of the body, leading to a loss of internal rhythmicity^31^. Night-shift workers are exposed to artificial light at night, associated with other unconventional synchronization signs, such as food intake, physical and emotional stress associated with the work performed, in addition to the alternation of work and rest shifts, which increase desynchronization. This long-term process can cause accelerated aging and increased risk of disease, which can be aggravated with age, when the biological clock adaptation decreases^32^.

Circadian rhythmicity and alcohol use are related to consumption levels. Different changes in circadian rhythms were observed after a single acute ingestion of alcohol, but also in alcohol abuse disorders and alcohol withdrawal. After a single acute alcohol ingestion, changes in biological rhythms are dosedependent, and reflected in melatonin and cortisol secretions and in core body temperature rhythms. These changes normalize the next morning and appear mainly in acute alcohol ingestion over 0.5g/kg. Circadian disturbance will depend on consumption levels and may persist after 3 to 12 weeks of abstinence^33^. The chronobiological effects of ethanol are related to melatonin suppression and inflammation, stress, free radical elimination, autophagy and cancer risk^34^.

The present study has limitations. First, this is a crosssectional study, which does not allow for causal relationships to be established. Second, the small sample size may have contributed to some non-significant results. Third, the small number of saliva samples collected (although each patient collected two samples at each time) reflected individual discrepancies in patient groups and may have avoided a larger variation in hormone levels.

Finally, sleep variables were obtained through a self-reported questionnaire and not through actigraphy.

## CONCLUSION

Night work can impact sleep, altering the circadian clock, disconnecting the environmental cycle (darklight) from the biological cycle (sleep-wake). In this context, adequate and good quality rest after night shifts is necessary to minimize the negative impacts on workers’ health and facilitate recovery of the HPA axis. Other aspects related to the workers’ health must be addressed preventively, in order to reduce the impact of night work, such as sleep hygiene (number of hours of sleep, sleep and wake up time, environmental conditions), as well as lifestyle habits such as smoking and alcohol consumption.
